# Suspected COVID-19 Reinfections at a Tertiary Care Center, Iowa 2020

**DOI:** 10.1017/ash.2021.34

**Published:** 2021-07-29

**Authors:** Takaaki Kobayashi, Mohammed Alsuhaibani, Miguel Ortiz, Katherine Imborek, Stephanie Holley, Alexandra Trannel, Alexandre Marra, William Etienne, Kyle Jenn, Oluchi Abosi, Holly Meacham, Lorinda Sheeler, Angie Dains, Mary Kukla, Paul McCray, Stanley Perlman, Bradley Ford, Daniel Diekema, Melanie Wellington, Alejandro Pezzulo, Jorge Salinas

## Abstract

**Background:** Coronavirus disease 2019 (COVID-19) is caused by the severe acute respiratory syndrome coronavirus 2 (SARS-CoV-2). SARS-CoV-2 RNA can be detected by real-time reverse-transcription polymerase chain reaction (RT-PCR) for several weeks after infection. Discerning persistent RT-PCR positivity versus reinfection is challenging and the frequency of COVID-19 reinfections is unknown. We aimed to determine the frequency of clinically suspected reinfection in our center and confirm reinfection using viral whole-genome sequencing (WGS). **Methods:** The University of Iowa Hospitals and Clinics (UIHC) is an 811-bed academic medical center. Patients with respiratory complaints undergo COVID-19 RT-PCR using nasopharyngeal swabs. The RT-PCR (TaqPath COVID-19 Combo kit) uses 3 targets (ORF1ab, S gene, and N gene). We identified patients with previous laboratory-confirmed COVID-19 who sought care for new respiratory complaints and underwent a repeated SARS-CoV-2 test at least 45 days from their first positive test. We then identified patients with median RT-PCR cycle threshold (Ct) values. **Results:** During the study period, 13,603 patients had a SARS-CoV-2– positive RT-PCR. Of these, 296 (2.2%) had a clinical visit for new onset of symptoms and a repeated RT-PCR assay >45 days from the first test. Moreover, 29 patients (9.8%) had a positive RT-PCR assay in the repeated testing. Ct values were available for samples from 25 patients; 7 (28%) had Ct values. **Conclusions:** In patients with a recent history of COVID-19 infection, repeated testing for respiratory symptoms was infrequent. Some had a SARS-CoV-2–positive RT-PCR assay on repeated testing, but only 1 in 4 had Ct values suggestive of a reinfection. We confirmed 1 case of reinfection using WGS.

Funding: No

**Disclosures:** None

